# Point Cloud Painting for 3D Object Detection with Camera and Automotive 3+1D RADAR Fusion

**DOI:** 10.3390/s24041244

**Published:** 2024-02-15

**Authors:** Santiago Montiel-Marín, Ángel Llamazares, Miguel Antunes, Pedro A. Revenga, Luis M. Bergasa

**Affiliations:** Department of Electronics, Universidad de Alcalá, 28805 Alcalá de Henares, Spain; angel.llamazares@uah.es (Á.L.); miguel.antunes@uah.es (M.A.); pedro.revenga@uah.es (P.A.R.); luism.bergasa@uah.es (L.M.B.)

**Keywords:** autonomous driving, sensor fusion, RADAR, camera, point cloud painting, object detection

## Abstract

RADARs and cameras have been present in automotives since the advent of ADAS, as they possess complementary strengths and weaknesses but have been underlooked in the context of learning-based methods. In this work, we propose a method to perform object detection in autonomous driving based on a geometrical and sequential sensor fusion of 3+1D RADAR and semantics extracted from camera data through point cloud painting from the perspective view. To achieve this objective, we adapt PointPainting from the LiDAR and camera domains to the sensors mentioned above. We first apply YOLOv8-seg to obtain instance segmentation masks and project their results to the point cloud. As a refinement stage, we design a set of heuristic rules to minimize the propagation of errors from the segmentation to the detection stage. Our pipeline concludes by applying PointPillars as an object detection network to the painted RADAR point cloud. We validate our approach in the novel View of Delft dataset, which includes 3+1D RADAR data sequences in urban environments. Experimental results show that this fusion is also suitable for RADAR and cameras as we obtain a significant improvement over the RADAR-only baseline, increasing mAP from 41.18 to 52.67 (+27.9%).

## 1. Introduction

The progress of autonomous driving (AD) technologies involves the development of perception techniques that enhance the scene-understanding capabilities of the vehicles. The perception stage of an autonomous driving stack (ADS) is in charge of interpreting the information from the sensors to understand the environment in which the vehicle is moving. This is accomplished in three steps: obtaining the information from onboard sensors, processing it, and interpreting the environment around the autonomous vehicle (AV). The information gathered by the sensors is presented in the form of point clouds, images or spectral signals.

The processing stage transforms these raw data into more refined information, such as the location, dimensions, and speed of the rest of the road users (pedestrians, cyclists, and other vehicles) or a semantic definition of the world in which every piece of information is assigned to an inanimated category (drivable road, vegetation or road obstacles). The literature reflects that learning-based algorithms have been predominant in this field since the advent of Deep Learning (DL). Once this elaboration is finished, it is possible to interpret the scene by adding a temporal and spatial coherence between the processed information in a timestamp and the processed information of past timestamps. This temporal–spatial association of information allows the system to predict which possible scenarios are likely to happen within the successive timestamps. The final output of the perception processing pipeline is used as the input of the upstream or downstream stages of the ADS.

Since the advent of Advanced Driver Assistance Systems (ADASs), conventional 2+1D RADAR and camera sensors have played a major role in the automotive industry as integral parts of these systems, such as Adaptive Cruise Control (ACC), Lane Departure Warning (LDW) or Assistance Emergency Braking (AEB). The strengths and weaknesses of both RADAR and camera sensors are complementary; thus, the mass implementation of ADAS systems has been fulfilled with this combination of sensors. Whilst RADAR provides sparse range, angle and velocity measurements, cameras provide pixel-level density representations with richer semantic features.

**Figure 1:**
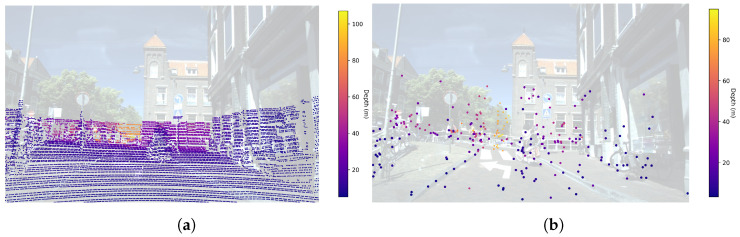
Comparison of a LiDAR point cloud (**a**) and 3+1D RADAR point cloud (**b**) projected to the image plane for an automotive scene of View of Delft dataset.

Nevertheless, in the transition from ADAS to AD technologies, academia and industry have shifted their main interests to perception systems based on LiDARs and cameras. LiDAR has replaced conventional 2+1D RADAR due to its better spatial and angular resolution and height measurement capabilities. In this situation, a new generation of high-resolution RADARs (3+1D) has emerged. Providing richer point cloud representations than their predecessors, and with a higher density of range, angle, height and velocity measurements, these new RADARs show potential for being reintroduced into the perception sensor suite in the context of AD applications. A visual comparison of the density of the point clouds obtained by a LiDAR and a 3+1D RADAR can be seen in Figure 1. Furthermore, the 3+1D RADAR provides 1D Doppler velocity measurements for each reflection, which are projected over the radial component of the detections. Doppler velocity be used to enhance downstream tasks such as object detection, linear velocity estimation and motion tracking. In contrast, LiDAR-based systems require the inference of any velocity component by analyzing the evolution over time of the objects of interest. The inclusion of radial velocity components enables the point cloud to be categorized into two distinct subsets: static and dynamic objects. The set of static objects can be leveraged to obtain 3D grid maps through the accumulation of multiple RADAR frames over time.

In this work, we aim to explore a geometrical data fusion approach to detect and classify objects as 3D bounding boxes in the environment surrounding the vehicle. Overall, the main contribution of this study is the implementation and evaluation of a multi-stage DL system for 3D object detection in automotive applications. The system utilizes data from 3+1D RADAR point clouds and monocular camera images, aligning with contemporary trends in sensor fusion in the perspective view (PV) with semantics. The primary goal is to achieve object detection and classification around the vehicle, combining the strengths of automotive RADAR and monocular cameras in a geometrical–sequential sensor fusion scheme. We summarize the rest of the contributions of our work as follows:

- We reformulate **PointPainting** [1] and adapt it to the domain of RADAR point clouds and camera images using **YOLOv8** [2] as the instance segmentation network and **PointPillars** [3] as the detection network, respectively.
- We design a set of rules based on heuristics to deal with the main disadvantage of the previous method, the *smearing* effect, to mitigate the propagation of errors from the segmentation stage to the detection stage.
- We evaluate our system in the novel **View of Delft** (VOD) [4] dataset, which includes 3+1D RADAR point clouds and a monocular camera image to perform sensor fusion for object detection, and we obtain significant improvements over the RADAR-only object detection method.

## 2. Related Works

Sensor fusion aims to fuse the data of different modalities or sensors, taking advantage of the synergies that can result from the combination of them to fulfill a specific task. In the context of AD, sensor fusion improves the robustness and reduces the uncertainty of perception systems. In this Section, we define how sensor fusion can be performed by answering four main questions: **what to fuse**, **where to fuse**, **when to fuse** and **how to fuse** [5]. Then, a set of selected methods based on camera and automotive RADAR is revised.

**What to Fuse?** In determining the fusion targets, RADAR presents a diverse array of modalities. These modalities span from the raw Analog-to-Digital Converter (ADC) data to point cloud representations, encompassing spectrograms and RADAR cubes. Each modality bears distinct advantages and limitations. Conversely, in the domain of cameras, representation diversity is diminished. Common modalities include RGB or gray-scale images for visible light cameras alongside infrared or thermal representations.

**Where to Fuse?** Spatial coherency must exist when fusing data from various sources. Data must be referenced to the same coordinate frame. To tackle this, there are two main options in RADAR–camera fusion: the perspective projection of the image plane or the bird’s eye view from space. Fusing in the image plane involves the projection of the RADAR tensors or point clouds via calibration matrices, forming sparse RADAR images. Fusing in the BEV plane involves the lifting of camera-based features inside a neural network since geometric projections under-perform in this task.

**How to Fuse?** Fusing two sensor modalities involves keeping spatial and temporal coherence or alignment. Temporal misalignment can occur when the latencies are not controlled or compensated. Spatial misalignment occurs when calibration methods are not accurate. Fulfilling these conditions, fusion is accomplished by means of fusion operators. These include transformation matrices for data-level fusions as well as additions, multiplication, concatenation and attention mechanisms for feature-level fusions.

**When to Fuse?** Depending on the stage of the perception pipeline, we can classify fusion schemes in these levels: object-level fusion, data-level fusion, feature-level fusion and any combination of them (hybrid-level fusion). Object-level fusion involves two separate independent methods that return their respective detections and a later fusion mechanism. In this case, Bayesian theory, Kalman filters and matching algorithms are used to associate detections spatially. The data-level fusion involves the creation of a new representation of the environment before any detection network or method, i.e., projecting the RADAR features to the image plane and concatenating those channels with the original RGB image, resulting in a different tensor that will feed a Neural Network (NN). The feature-level fusion involves the combination of RADAR feature maps and camera feature maps inside an NN applying any fusion operator (keeping spatial coherence). Then, hybrid approaches can make combinations of any of the previous ones applying different fusion operators.

**Methods.** Having reviewed the main components of sensor fusion, it is possible to review the recent literature in terms of RADAR–camera fusion for perception.

nuScenes [6] has become the dataset of reference for AV applications and has been the first big-scale benchmark to introduce conventional (2D+1) RADAR data. One of the first methods ranked in nuScenes for the camera and RADAR Detection Track was **CenterFusion** [7]. Nabati and Qi propose a feature-level fusion scheme with two main branches. The imaging branch uses **CenterNet** [8] as the backbone. The RADAR branch opts for a pillar rendering mechanism, which is similar to KPConv. The head is split into two parts: primary and secondary. The primary head obtains preliminary 3D bounding boxes. Pillar features and preliminary boxes are associated through a frustum-based mechanism, and a RADAR feature map is generated. Image and RADAR feature maps are concatenated and are the input to the secondary head, which refines the preliminary proposals. They performed a performance comparison in which the camera-only KPConv scored an NDS = 0.328 and the fusion of both sensors scored an NDS = 0.453, which is an improvement of 0.125 due to the fact including RADAR data information.

Nobis et al. [9] proposed a method to fuse RADAR and camera information in the image plane, extending the image with RADAR channels that include range, velocity and RCS information. The method is built based on **RetinaNet** [10] with two branches. The camera image follows a VGG [11] backbone, and the RADAR branch applies max pooling operations to preserve the maximum information possible when the dimensionality is reduced as it is a sparse image. Then, feature maps from RADAR and image branches are concatenated at multiple scales. An FPN and a detection head end the architecture. The method introduced a novel augmentation method called *BlackIn*. *BlackIn* shut down the neurons at the input of the camera image, so the network must rely on the RADAR channels to obtain the information.

A remarkable network that opted for a data-level approach is **RadSegNet** [12]. The method uses a 2D detector for oriented bounding boxes. In this case, a BEV grid map is generated from the RADAR point cloud. The grid is augmented with semantic information coming from the camera. A 2D semantic segmentation network is applied to the image. Then, a semantics-to-RADAR module projects the information from the image plane to the BEV grid map. The final grid map is composed of multiple channels: the grid map, point-based feature maps and semantic maps, leading to a 22-dimensional tensor. The architecture for semantic segmentation is a DeepLabV3+ with a ResNet-101 backbone trained in CityScapes [13] due to the absence of semantic labels in RADAR–camera-based datasets. The architecture for object detection is based on RADAR, treating detection as a per-pixel task, which was trained on the Astyx dataset [14].

Revisiting the existing literature and the methods that have been previously explained, it is possible to conclude that the future direction of sensor fusion is shifting from object or data-level fusion schemes to relying on BEV fusion mechanisms for perception [15]. Combining both modalities in the bird’s eye view space leads to the design of novel mechanisms to either lift camera features to 3D space solely [16] or assisted by RADAR [17] via attention mechanisms.

Table 1 summarizes the methods revisited in this section. All methods are based on conventional 2+1D RADAR, and neither of them fuses 3+1D RADAR and image semantics in the image plane as our proposal.

## 3. Method

**Figure 2:**
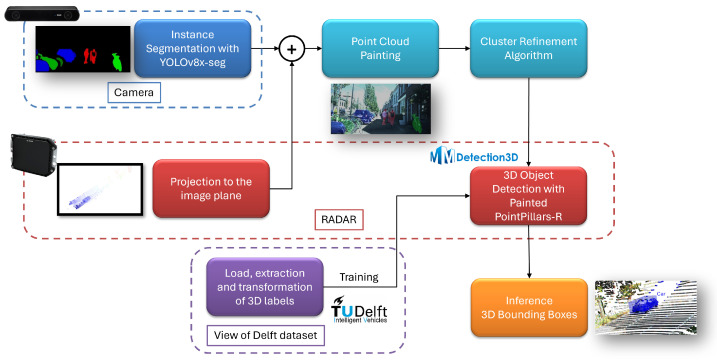
Overview of the complete pipeline.

The problem dealt with is 3D object detection, which is typically formulated in the literature as the regression and classification of 3D-oriented bounding boxes that enclose objects of interest for the autonomous navigation task. To address this task, we propose a pipeline based on [1] to perform 3D object detection based on painted automotive RADAR point clouds via segmented camera images. The method is composed of four stages: instance segmentation, point cloud painting, rule-based cluster refinement and 3D object detection. A diagram representing the whole pipeline is shown in Figure 2.

### 3.1. Two-Dimensional (2D) Instance Segmentation

The first step of the framework consists of the application of a 2D instance segmentation network to the camera image to obtain the semantic category of the objects of interest that are present in the scene. We employ YOLOv8x-seg as an instance segmentation neural network to mitigate the performance drop that occurs due to the absence of specific semantic labels and a domain shift in the dataset studied, as this network has been extensively pre-trained on the COCO dataset [18].

YOLOv8 formulates the instance segmentation task as a “segmentation-by-detection” problem. It first detects an object of interest and then adjusts a binary mask that indicates which pixels in the image belong to that corresponding instance. The structure of the network is as follows: a CSP-Darknet53 backbone [19], a PAFPN-based neck, a decoupled head for detection and Proto heads for upscaling the images to their original size and extracting the segmentation mask per instance. Therefore, the output of this network is a set of instances where each instance is characterized by a bounding box, a semantic category, an objectness score that ranks from 0 to 1, which are output by the decoupled detection head and a binary segmentation mask output by the Proto segmentation heads. The main innovation in this stage is the use of instance segmentation networks, which focus on VRUs, instead of semantic segmentation networks as were in PointPainting [1] in which also background elements are segmented.

**Figure 3:**
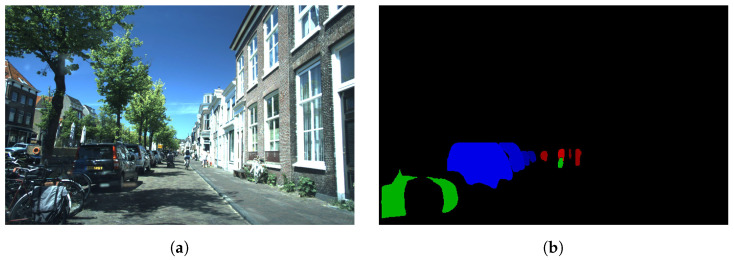
Monocular camera image (**a**) and instance map highlighting pedestrians, bicycles and cars (**b**) for frame 03456 in the View of Delft dataset.

We run inference for each image, and after that, we apply the following processing. We filter the masks that belong to the classes: vehicle, person and bicycle. For each filtered instance, we multiply its segmentation mask by its objectness score. We sum all the masks, encoding each one of the semantic categories in an image channel. Finally, we saturate the channels to 1 to cover the edge case in which various masks within the same semantic categories overlap in the same area of the image. We call this representation the instance map, and an example can be seen in Figure 3. In this figure, an urban scenario is represented. In the left part of the image, multiple bicycles (green) and cars (blue) are segmented correctly. It can be noticed that the bluer tone is decreasing to distance, meaning that the confidence in the segmentation network is decreasing following the same pattern. In the center of the road, multiple pedestrians are correctly segmented (red). As previously stated, it can be seen that cyclists are segmented as a person (red) and bicycle (green) independently.

### 3.2. Point Cloud Painting

To perform sensor fusion between camera and RADAR data coherently, both sources of data must be spatially aligned. Painting a point cloud implies fusing features that are located in pixels of an image plane into their corresponding points in 3D space. Therefore, we follow [1] to perform data fusion. Nevertheless, we apply painting with both RGB images and instance maps.

We apply a homogeneous transformation from the RADAR coordinate frame to the image coordinate frame and project all RADAR points into the image plane via Equation (1), where $P_{XYZ1}$ is an $R^{4\times N}$ matrix of *N* homogeneous points in the RADAR coordinate frame, $T_{radar\;to\;cam}$ is a homogeneous transform from RADAR to the camera ($R^{4\times 4}$), $R_{0,\;rect}$ is a rectification matrix ($R^{4\times 4}$), $P_{2}$ is a pseudo-intrinsic parameters matrix for stereo systems ($R^{3\times 4}$) and $P_{UVW}$ is the output result, a matrix.

(1) $$P_{UVW}=P_{2}\cdot R_{0,\;rect}\cdot T_{radar\;to\;cam}\cdot P_{XYZ1}$$

Once the points are located in the image plane, we can establish a point–pixel correspondence for all the elements of the point cloud. After this process, we obtain a painted point cloud whose dimensionality has been increased, $R^{N\times C+3+3}$, where *C* is the original number of features in the RADAR point cloud, 3 additional channels encode the RGB color of the image, and the last three channels encode the semantic category assigned to that point at the instance segmentation stage. The inclusion of color RGB channels in the painting mechanism is the main innovation of this stage.

### 3.3. Cluster Refinement

The main weakness of the painting method is the *smearing* effect. It is an effect that occurs when the segmentation of an object of interest is imperfect and produces a trace of colored or segmented points in the angle bin in which this object is projected. Objects of interest affected by this effect will show a different spatial distribution than objects that are correctly segmented. This potentially leads to the propagation of errors from the segmentation stage of the pipeline to the detection stage.

We identify four potential causes that could lead to the appearance of *smearing* phenomena: the quality imperfection of the segmentation masks, the calibration and temporal misalignment between the point cloud and the image, the absence of motion compensation and the propagation scheme of RADAR reflections, since multiple reflections can appear in the same angle bin.

As the instance segmentation networks allow us to propagate the instance concept, it is possible to discern which RADAR points belong to which segmented instance. We design a set of heuristic rules to identify objects affected by smearing and mitigate the propagation of erroneous paintings into the detection stage. We approach this problem by analyzing the radial dispersion of the detected objects, Δr=max(r)−min(r). We obtain the radial distance to all points in an instance of interest and determine their difference. If the dispersion is greater than a certain threshold, the object is considered to be affected by *smearing*. To set these thresholds, we double the anchors set by the object detection network that will be used in the detection stage for the semantic categories we are coping with. The reason behind doubling the anchors and taking them as thresholds is to detect the most affected cases in which *smearing* will contaminate the data fusion process. The doubled anchors chosen are 7.8 m for the *Car* category, 3.52 m for the *Cyclist* category and 1.6 m for the *Pedestrian* category.

**Figure 4:**
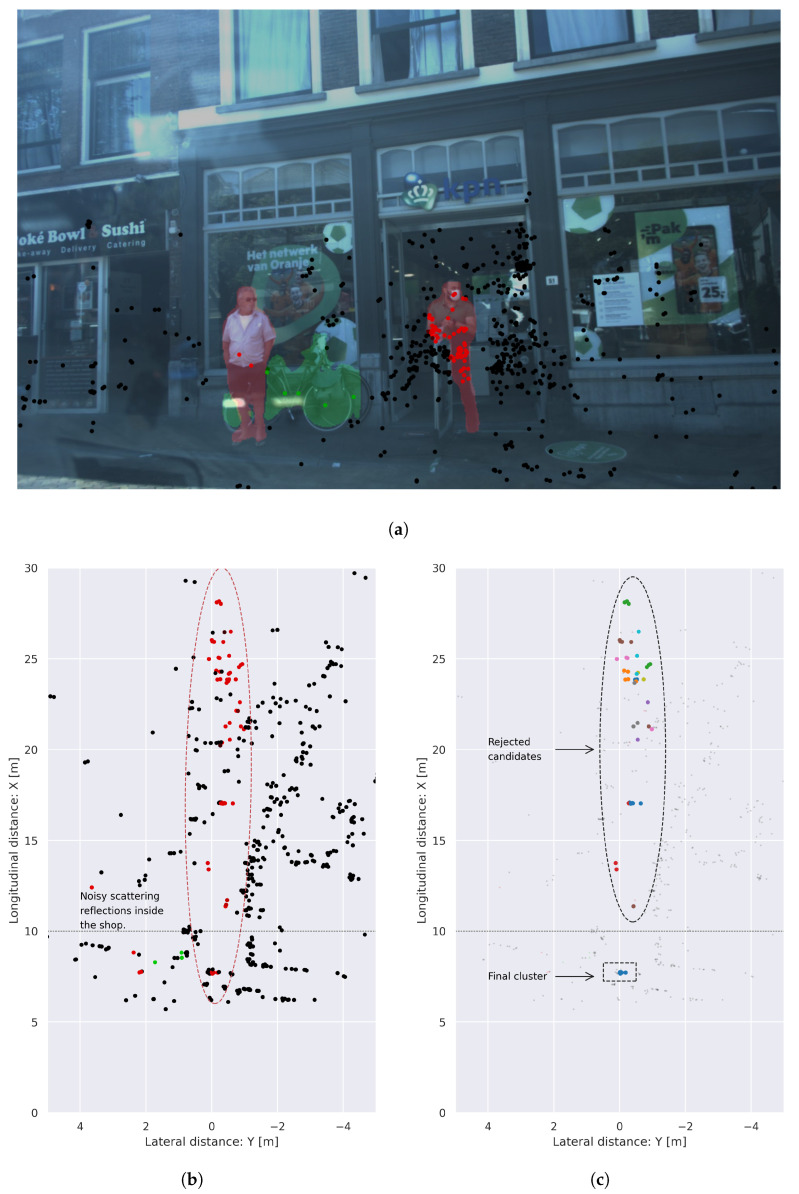
Example of cluster refinement algorithm applied in frame 00125 of the View of Delft dataset. A large set of reflections produced by scattering is generated inside the shop and projected into the pedestrian standing at the door in image (**a**), producing a large smearing that can be seen in (**b**). The cluster refinement algorithm detects the radial dispersion and corrects the probabilities assigned to the points in the point cloud (**c**).

Once an object is considered to be affected by smearing, we determine if the object is dynamic or static by analyzing the presence of points with a component of compensated radial velocity in absolute value, $|V_{r\;comp}|\geq$ 0.3 m/s. If an object is considered to be dynamic, we apply DBSCAN [20] clustering over the radial velocity features and we extract the cluster with more detections and mean velocity different from zero. If an object is considered static, we apply DBSCAN clustering over the spatial dimensions, and we retain the closest cluster in range, i.e., the first one encountered in the line of sight. An example of the application of this cluster refinement can be seen in Figure 4. In this qualitative scenario, the smearing effect is present as a large set of reflections that range from 10 to 30 m, which is produced due to the effect of RADAR scattering and noisy propagation inside a shop. As a pedestrian is standing in front of the shop door and multiple reflections are lying behind in the same radial direction, various points are incorrectly assigned to this instance when the painting procedure is performed. Applying the cluster refinement algorithm, we can identify the blob of points that belong to the object and filter noise that is not propagated into the following stage of the system.

### 3.4. Three-Dimensional (3D) Object Detection

The final step of the pipeline consists of the application of a 3D object detection network over the painted point cloud, by using a version of PointPillars [3] which is adapted for RADAR data.

**Figure 5:**
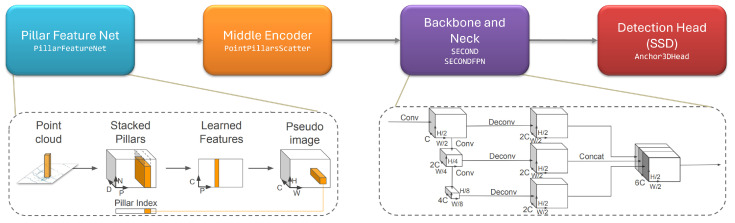
Overview of the PointPillars architecture. Adapted from [3].

The architecture of PointPillars can be seen in Figure 5 and is structured as follows: a data preprocessing module, which is responsible for voxelizing point clouds with a random sampling strategy to augment the training data; a Pillar Feature Net extracts features per pillar using the arithmetic mean of points and their offset from the center; a Middle Encoder converts learned features to a sparse pseudo-image, facilitating subsequent processing; then, the architecture comprises a backbone, neck, and head, following the typical scheme of a 2D object detection network. The backbone is a convolutional network, SECOND [21], producing features with decreasing resolutions. The neck performs upsampling and concatenates features from different resolutions. The head incorporates prior information about bounding box sizes and generates anchors for pedestrians, cyclists, and cars. Object detection is treated as a matching problem using Intersection over Union (IoU), with additional regression tasks for height and elevation, forming a complete 3D object detection network.

The main innovation in this stage is the adaptation of PointPillars to fit RADAR data. For this purpose, we perform the following modifications to the previously explained scheme:

- We adapt the point cloud range to the dataset we are coping with, which is explained in Section 4.1. We modify as a consequence the anchor generation range.
- We increase the voxel size. Since the RADAR sensors are placed in the front bumper of the vehicle, we explore the height range of $\left[-2,\;3\right]$ m.
- We reduce the maximum number of points per voxel. Since RADAR point clouds are sparser than LiDAR ones, we decrease this threshold from 32 to 10 points.
- We delete data augmentation that deforms or rotates point clouds, since the RADAR information would become physically incoherent.
- We modify all the components that load point clouds and adapt them to the new dimensionality from LiDAR ($R^{N\times 4}$) to RADAR ($R^{N\times 7}$) or Painted RADAR ($R^{N\times 13}$).

With these modifications taken into consideration, PointPillars can perform inference and detect 3D objects in modified and painted RADAR point clouds.

## 4. Experiments and Results

### 4.1. Dataset

The experiments are carried out on the **View of Delft** (VoD) dataset. It was released in Q2 2022 and includes a collection of urban sequences recorded in Delft, The Netherlands. These sequences lead to 8682 individual frames with an official training–validation–testing split of 5139, 1296, and 2247, respectively. Each frame contains readings from multiple sensor modalities, of which LiDAR, RADAR, camera and their respective calibration and transform matrices are of particular interest for the validation of this work. Moreover, annotations for 2D and 3D object detection tasks are provided.

Delving into the sensor readings, LiDAR point clouds are captured at 10 Hz by a Velodyne HDL-64 S3. Every LiDAR point cloud is a vector $L_{PC}\;\epsilon \;R^{N\times 4}$, where *N* denotes the number of points in a point cloud and every point is a tuple $P_{L}=\left(x,\;y,\;z,\;i\right)$ that represents its spatial location $\left(x,\;y,\;z\right)$ and its reflection intensity $\left(i\right)$. RADAR is captured by a ZF FRGen21 3+1D sensor at 13 Hz. Analogously, a RADAR point cloud is a vector $R_{PC}\;\epsilon \;R^{N\times 7}$ with *N* points and seven features per point. Then, $P_{R}=\left(x,\;y,\;z,\;vr,\;vr_{comp},\;\sigma ,\;t\right)$, where $\left(x,\;y,\;z\right)$ is the spatial location of the reflection, $\left(vr,\;vr_{comp}\right)$ is the radial velocity without and with ego-motion compensation, respectively, $\left(\sigma \right)$ is the RADAR cross-section and $\left(t\right)$ is a time component that allows the accumulation of multiple timestamps in a single point cloud. Lastly, a stereo vision system provides a single color image in the form $I\;\epsilon \;Z^{3\times H\times W}$, where the height and width are H=1216 and W=1936, respectively.

The annotations are provided in a KITTI-like style and are provided for each LiDAR scan in the dataset and cover an area of 50×50 m on both lateral and longitudinal axes. A set of 15 semantic classes is contained within the dataset with a focus on Vulnerable Road Users (VRUs), such as *pedestrians* and *cyclists*. For evaluation purposes, we map the original classes to *Pedestrian*, *Car* and *Cyclist* to follow the KITTI evaluation protocol.

### 4.2. Implementation Details

The components of the detection pipeline are trained and/or executed on an Intel i9-12700 CPU and an NVIDIA RTX4090 24 GB GPU; therefore, the training and inference times are reported concerning these hardware specifications. The implementation of the instance segmentation network is based on the ultralytics (Ultralytics on GitHub: https://github.com/ultralytics/ultralytics, accessed on date: 27 December 2023) package and the 3D object detection is implemented modifying PointPillars for LiDAR at mmdetection3d (mmdetection3d on GitHub: https://github.com/open-mmlab/mmdetection3d, accessed on date: 27 December 2023).

### 4.3. Performance on 3D Object Detection

We evaluate the performance of the pipeline on the 3D object detection task. For this procedure, we train **PointPillars** models following a KITTI standard training procedure: 80 epochs, where the learning rate is configured using around an initial value of $\eta =1\times 10^{-3}$ and is modified every epoch using a learning rate scheduler: **Cosine Annealing** with two stages. The first stage covers from the beginning of the training to epoch 32 and increases from $10^{-2}$ to $10^{-3}$ in a linear trend. The second stage covers from epoch 32 to the end of the training in epoch 80 and decreases η from $10^{-3}$ to $10^{-7}$ following Equation (2). The optimizer is AdamW, which is an adaptive momentum optimizer with weight decay.

(2) $$\eta_{t}=\eta_{min}+\frac{1}{2}\left(\eta_{max}-\eta_{min}\right)\left(1+\cos\left(\frac{T_{cur}}{T_{max}}\pi \right)\right)$$

As for the loss function, we employ a weighted sum of three different components: focal loss [10], smoothed L1 [22] and cross-entropy, as Equation (3) states.

(3) $$L=\alpha_{1}\cdot L_{FL}+\alpha_{2}\cdot L_{L1_{smooth}}+\alpha_{3}\cdot L_{CE}$$

Focal loss is in charge of bounding box classification. It aims to address class imbalance during training, adding a modulating term to cross-entropy loss to focus on hard misclassified examples, following Equation (4). For our experiments, $\alpha_{1}=1.0$ and γ=2.0.

(4) $$L_{FL}=-{\left(1-p_{t}\right)}^{\gamma }\cdot \log p_{t}$$

Bounding box regression is dealt with a Smooth L1 term that uses a square penalty term term if an error falls below beta and an L1 otherwise. It prevents exploding gradients and follows (5). For our experiments, we set $\alpha_{2}=2.0$ and β=1.0/9.0.

(5) $$L_{L1_{smooth}}=\left\{\begin{array}{cc} 0.5\cdot {\left(x_{n}-y_{n}\right)}^{2}/\beta & \mathrm{if}\;|x_{n}-y_{n}|<\beta \\ |x_{n}-y_{n}|-0.5\cdot \beta & \mathrm{otherwise} \end{array}\right.$$

Finally, bounding box orientation is estimated formulating the problem as a classification, where the goal is to categorize each box into predefined bins. For our experiments, we set $\alpha_{3}=0.2$.

We use Mean Average Precision (mAP) and Average Orientation Similarity (AOS) as metrics, which are defined as follows:

- Mean Average Precision (mAP) assesses the performance on axis-aligned 3D bounding box detection and weights the Average Precision (AP) results across all *n* categories. It is defined in Equation (6).
  (6) $$mAP=\frac{1}{n}\sum_{k=1}^{k=n}AP_{k}$$
  Average Precision for a single category is defined at Equation (7). It is a discrete evaluation over the recall curve in which detections are iteratively assigned to a ground truth object and assigned as true positive (TP) if their Intersection over Union (IoU) is more than a certain threshold. Multiple detections over a single ground truth object are considered false positives.
  (7) $$AP=\frac{1}{40}\sum_{k=0}^{k=40}Precision\left(\frac{k}{40}\right)$$
  Concerning the selection of IoU thresholds for every category, we follow the recent literature for RADAR 3+1D detection [4], which defines the minimum IoU thresholds necessary to consider an object as TP: IoU =0.5 for *Car* category and IoU =0.25 for *Pedestrian* and *Cyclist* categories.
- Average Orientation Similarity (AOS) assesses the performance of estimating the 3D orientation of the bounding box. Moreover, it is related to the recall at the object detection stage, from which multiple thresholds are set to perform a discrete evaluation over the recall curve, which is expressed as shown in Equation (8).
  (8) $$AOS=\frac{1}{40}\sum_{k=0}^{k=40}\max s\left(\frac{k}{40}>r\right)$$
  It is derived from a normalized variant of cosine similarity, $s\;\epsilon \;\left(0,1\right)$, and computes the error from the difference between an estimated and ground-truth orientation for every 2D detection in the image plane, as Equation (9) states.
  (9) $$s\left(r\right)=\frac{1}{D(r)}\cdot \sum_{i}\frac{1+\cos\Delta_{\theta }^{\left(i\right)}}{2}\cdot \delta_{i}$$
  where D(r) is the set of detections for a certain recall value *r* and $\Delta_{\theta }^{\left(i\right)}$ is the difference between angle θ estimation and ground truth for a matched detection *i*. Additionally, $\delta_{i}$ acts as a penalization mechanism: $\delta_{i}=1$ if IoU_*i*_ $\geq IoU_{min}$ or $\delta_{i}=0$ otherwise.

The comparison will be performed between **PointPillars** for RADAR, which we will refer to as **PointPillars-R**, and the colorized version, which we will refer to as **Painted PointPillars-R**. The first one will serve as a baseline to compare with the latter, our approach. Moreover, we will experiment with the temporal aggregation of RADAR point clouds among frames, and we will train for each of the versions of the VoD dataset: single frame, 3 frames and 5 frames. As a remark, the metrics will be obtained over the validation set of VoD.

Table 2 shows the results of training the baseline **PointPillars-R** over the validation set of the VoD dataset. The table is split among three different criteria: metric, difficulty and categories. Among metrics, from left to right: mAP in the BEV plane, mAP in the 3D space, and AOS. Among difficulties: easy, medium and hard, depending on the size of the bounding box in the image plane and its occlusion level (moreover, in KITTI, the difficulty includes the level of truncation as an additional criterion, but this attribute is not labeled in the View of Delft dataset). Among categories, the three evaluated ones are *Car*, *Pedestrian* and *Cyclist*.

The performance results of the network indicate that RADAR-based methods have difficulties extracting bounding boxes for the *Car* and *Pedestrian* categories. Poor spatial resolution leads to scarce reflections in the targets of interest and difficulties in estimating the shape of the objects. The network shows successful performance in the *Cyclist* category, ranging from 63.25 3D AP40 in easy difficulty and single frame to 73.15 in easy difficulty and five accumulated frames. This is an atypical situation in automotive datasets, where VRUs are detected with difficulty. This may be due to the appearance of radial velocity and RCS as additional channels in the RADAR point clouds. After all, these features can be an excellent source of information to distinguish the few reflections that fall within cyclists from the static or different-in-terms-of-reflectivity background. Moreover, comparing single-frame and multi-frame versions, the increment between 1 and 3 frames is more notorious than the one between 3 and 5 scans for 3D AP40 across all categories. However, a rising trend among frame versions is perceived. Then, the network is taking advantage of the temporal aggregation of scans.

After this, we evaluate the performance of our proposal **Painted PointPillars-R** following the same schema as the baseline, whose results can be seen in Table 3. We train the single-frame and multi-frame versions following the same procedure. At a glance, it is observed that there is a clear improvement in both ways: comparing version to version with the baseline and comparing between the number of scans used for temporal aggregation. For example, in the case of 3D AP40 for *Cars*, the metrics increase for the baseline: 32.45 vs. 30.42 in medium difficulty; and they increase with respect to temporal aggregation within the proposed architecture: from 32.45 in single-frame mode to 39.64 in five-frames aggregation mode.

It is observed that for the proposed version, mAP40 for all classes increases in both spaces, BEV and 3D, with respect to the baseline results. This means that the semantic information not only improves the height estimation but also contributes to the estimation of the width and length of the objects of interest, which leads to an overall better shape estimation of the bounding box.

**Figure 6:**
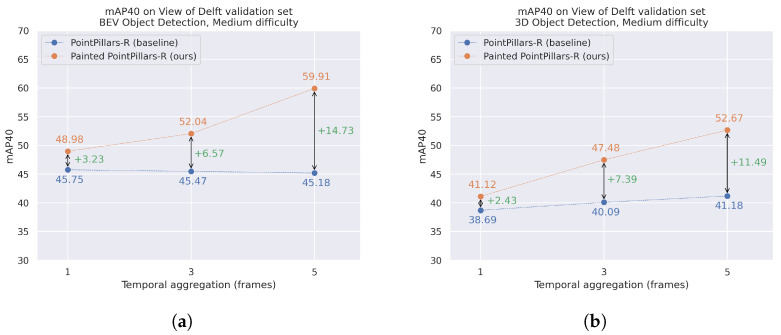
Comparison on Mean Average Precision (40 points) between PointPillars-R and Painted PointPillars-R over the View of Delft dataset validation set for bird’s eye view (**a**) and 3D object detection (**b**).

Lastly, as the main objective of this pipeline is to perform 3D object detection, we compare the results on 3D mAP (medium difficulty) for **PointPillars-R** (in blue dots) and **Painted PointPillars-R** (in orange dots) in Figure 6. In the X-axis, we plot the three different versions depending on the temporal aggregation horizon (number of frames). In the Y-axis, we plot the metrics extracted from taking the average of all categories in medium difficulty. We remark on the incremental improvement between the baseline and our proposal for all temporal-aggregated versions of the dataset: ΔmAP $=\left(+3.23,\;+6.57,\;+14.73\right)$ for BEV space and ΔmAP $=\left(+2.43,\;+7.39,\;+11.49\right)$ for 3D space. Moreover, it is shown that this delta increases with respect to the temporal aggregation of scans.

### 4.4. Runtime Performance

In this section, an analysis of the runtime performance will be conducted. We measure the execution time of the components of the pipeline following the experimental setup described in Section 4.2. We will test the inference time of all the components separately, performing cycles of 1000 executions and time measurements for each of them.

Firstly, we will test the inference time of the segmentation network, **YOLOv8-seg**. For this study, we will compare all versions: from *nano* (n) to *extra-large* (x). Three processes are taken into account: preprocessing (resize operation), inference and postprocessing (NMS). We use version **x** in the final pipeline as it offers the best performance, and as VoD does not have semantic labels, we minimize the impact of the domain shift. Table 4 reflects the results. Processing steps consume a fixed amount of time, while inference time increases from 1.7 ms in the lightest version to 6.8 ms in the heaviest one.

Subsequently, the following steps in the pipeline are the **PointPainting** geometric fusion and the cluster refinement algorithm. The first one contains an *upsampling* operation, so we measured a mean time of 7.28 ms with a standard deviation of 0.25 ms. The latter one contains several runs of DBSCAN clusterings. We measured a mean time of 14.3 ms and a standard deviation of 0.36 ms. Then, we test the inference time of **PointPillars-R** and **Painted PointPillars-R** for the three versions of the VoD dataset. The results are reported in Table 5. It is observed that all models infer at an approximate time of 6 ms, and there are no variations among the baseline and our proposal or among dataset versions.

Finally, choosing **YOLOv8x-seg** and **Painted PointPillars-R** (the five frames temporal-aggregated dataset version) as components of our proposed framework, the total inference time is **35.94 ms** or 27.82 Hz. Therefore, the solution is suitable for real-time applications in the context of automotive perception systems, even when there is room for further optimization in future works.

### 4.5. Comparison with PointPainting for LiDAR

To set a fair comparison with LiDAR-based methods, we perform an experiment in which we train PointPillars for LiDAR point clouds. We follow the same training and evaluation procedures previously explained in Section 4.3, and transfer learning from other datasets is not performed. We name these methods **PointPillars-L** and **Painted PointPillars-L**, respectively. Table 6 shows the performance in medium difficulty for the validation set of the VoD dataset, in which LiDAR-based methods outperform (69.40 and 73.14 mAP) RADAR-based methods (41.18 and 52.67 mAP). A remarkable fact is that the camera contribution is far more significant from non-painted to painted methods in the RADAR domain (+11.19 mAP) than in LiDAR (+3.74 mAP).

### 4.6. Ablation Study

In this section, we want to remark on the contribution of every single component of the system: from the baseline, **PointPillars-R**, to the proposed system, **Painted PointPillars-R** in an accumulated five-frames settings. For all the experiments, we follow the training and evaluation procedures previously explained in Section 4.3.

Table 7 shows the results of the ablation study. Starting from the baseline (38.69 mAP), the inclusion of the painting mechanism implies a significant performance boost (+2.17 mAP). Then, the cluster refinement algorithm allows noise filtering and contributes a minor improvement. Finally, the accumulation of frames contributes another significant performance boost. Comparing with the results in Section 4.3, it can be observed that the painting mechanism improves also its contribution concerning the number of frames.

### 4.7. Quantitative Results

This section presents qualitative results for the model **Painted PointPillars-R** in the version of five frames accumulated for the VoD dataset.

**Figure 7:**
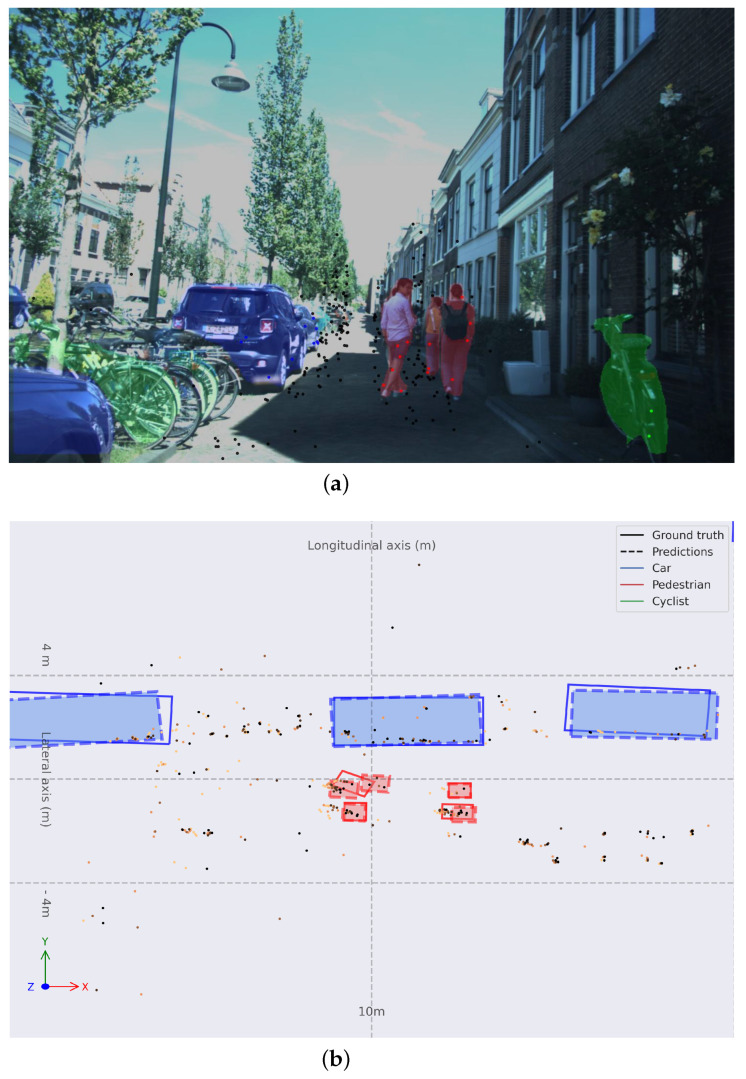
(**a**) Monocular image with colored instance map and RADAR point cloud projected. (**b**) Object detection in BEV space for frame 03970 with **Painted PointPillars-R**. Bounding boxes are: solid for ground truth and dashed and filled for predictions; red for *Pedestrian*s, green for *Cyclist*s, and blue for *Car*s. Older points are lighter orange and more recent points are darker orange.

In Figure 7, a common use case within the dataset is presented. A narrow urban street with one single direction with vehicles parked at the left hand of the ego vehicle and pedestrians walking in front of the ego vehicle on the drivable road. In Figure 7b, a trace can be observed behind the pedestrians, showing that are moving frontwards. The algorithm can perceive correctly in this case. Few points with the semantics of *Pedestrian*s are enough to propose bounding boxes at coordinates $\approx \left(10,0\right)$ m and $\approx \left(12,0\right)$ m. *Car*s at long range are perceived correctly.

**Figure 8:**
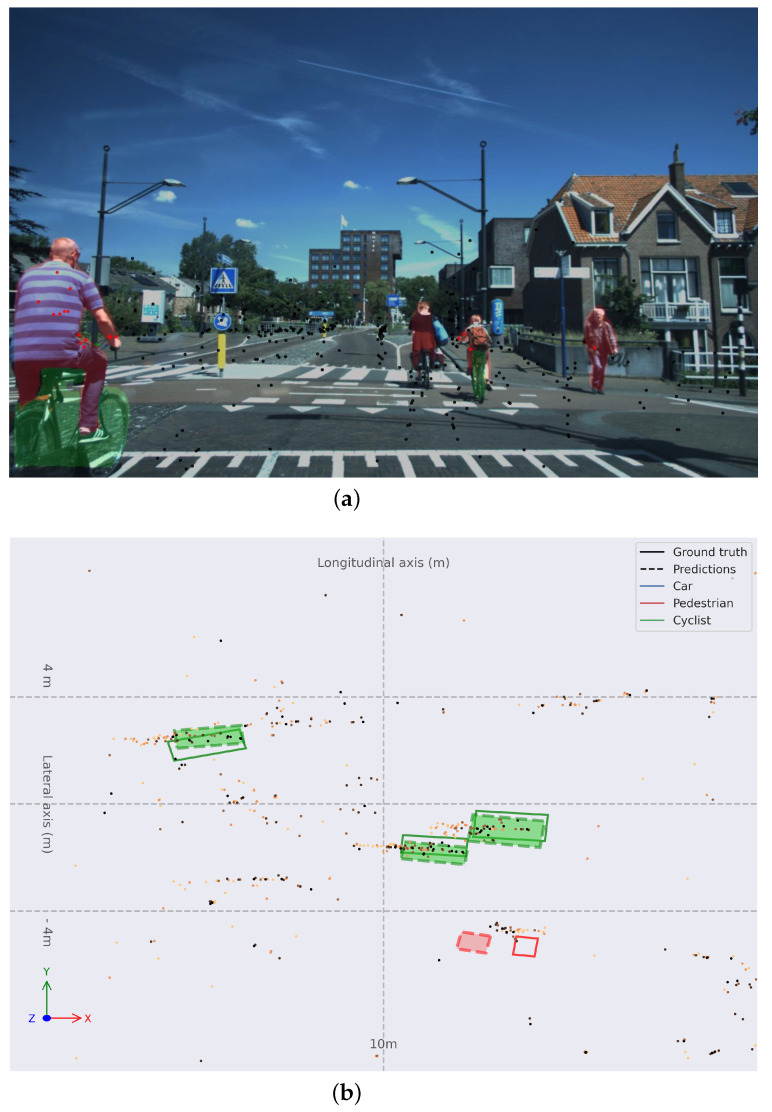
(**a**) Monocular image with colored instance map and RADAR point cloud projected. (**b**) Object detection in BEV space for frame 04362 with **Painted PointPillars-R**. Bounding boxes are solid for ground truth and dashed-and-filled for predictions; red for *Pedestrian*s, green for *Cyclist*s, and blue for *Car*s. Older points are lighter orange, and more recent points are darker orange.

Figure 8 justifies the good performance on the *Cyclist* category. One of the main characteristics of the VoD dataset is that this category is well represented and there are frames in which it is the only category present. This is an atypical use case in automotive datasets that takes place due to the location in which VoD is recorded is The Netherlands.

**Figure 9:**
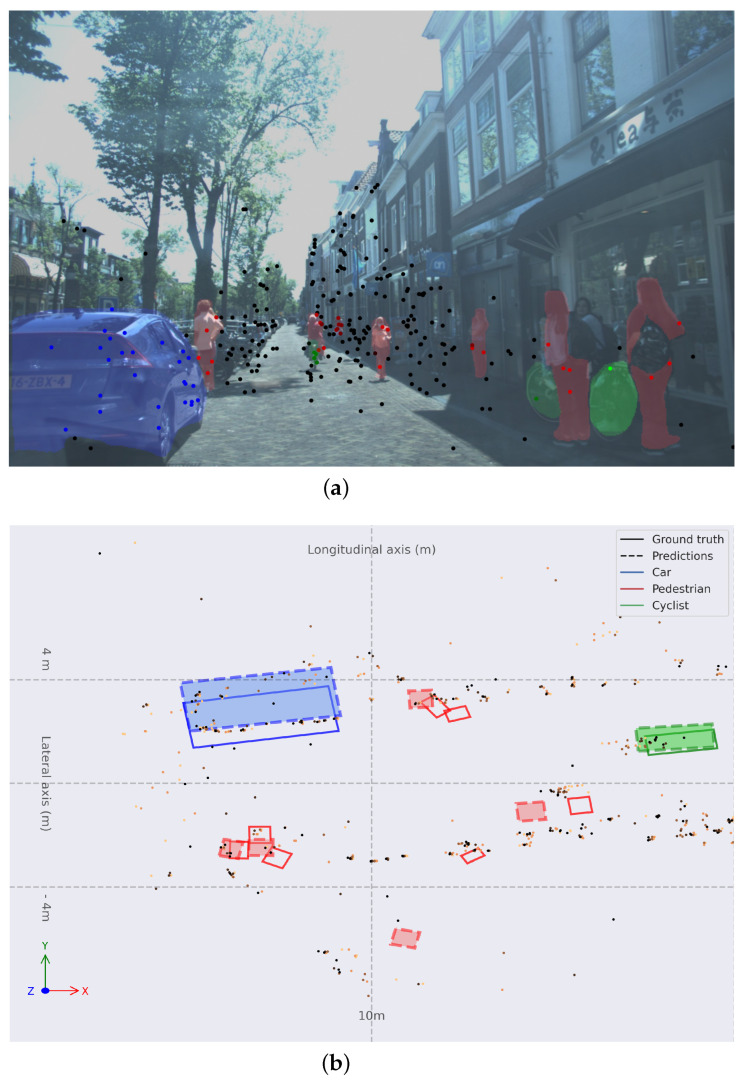
(**a**) Monocular image with colored instance map and RADAR point cloud projected. (**b**) Object detection in BEV space for frame 00180 with **Painted PointPillars-R**. Bounding boxes are solid for ground truth and dashed-and-filled for predictions; red for *Pedestrian*s, green for *Cyclist*s, and blue for *Car*s. Older points are lighter orange, and more recent points are darker orange.

Figure 9 shows a crowded environment with multiple *Pedestrian*s. Most of them are detected correctly. It is remarkable to notice that most *Pedestrian* instances have few points inside their ground-truth bounding box, so the network may show difficulties. The *Cyclist* and *Car* are detected correctly. In the case of the *Cyclist*, a trace in the point cloud can be observed. Most of the *Cyclist*s in this dataset are moving objects, so the accumulation of the frame plays a vital role in inferring the position of these VRUs.

## 5. Conclusions and Future Works

In this paper, we validate the performance of a sensor fusion algorithm for 3D object detection which originally was designed for LiDAR and a camera in the domain of 3+1D RADAR and camera data fusion in the View of Delft dataset.

The research introduces compelling insights, particularly highlighting the potential of RADAR–camera fusion for 3D object detection. This fusion significantly outperforms the original RADAR-only baseline across all classes, showcasing the efficacy of projection-based fusion methods. The study also emphasizes the positive impact of temporal information aggregation on neural networks, elevating their precision.

Notably, the analysis spotlights the unexpected performance boost in detecting cyclists, which is a crucial aspect for the safety of vulnerable road users. The study challenges conventional notions, indicating that an increased representation of cyclists enhances network performance, which can be potentially attributed to the unique information provided by radial velocity and RCS features.

Experiments were conducted to compare the performance of RADAR-based PointPainting and LiDAR-based PointPainting. Two main conclusions were drawn. Firstly, LiDAR outperforms RADAR due to the higher density of reflections in the point cloud. As a consequence, more research is needed to fully utilize RADAR data and shorten this gap. Unique features such as RCS and 1D Doppler velocity have not received much attention in the existing literature. Both the inclusion of these components and the investigation of RADAR-specific network architectures could help to achieve LiDAR-like performance for object detection networks. Secondly, it is observed that the contribution of the camera sensor to the RADAR–camera fusion schemes is comparatively higher than that of LiDAR-camera fusion methods, as the improvements from their respective baselines methods are +11.19 mAP and +3.74 mAP. This experiment supports the claim that RADAR and camera sensors have complementary strengths and weaknesses, making them suitable for an intelligent transportation sensor suite for perception.

However, challenges emerge, such as the negative impact of eliminating data augmentation on convergence during training. This underscores the importance of devising new augmentation techniques that consider the physical nuances related to radial velocity. Additionally, the study sheds light on the sensitivity of the validation set, particularly in urban scenes, necessitating improvements for generalization with DL-based techniques.

As a result of this work, we identify future lines of research in the domain of RADAR and camera fusion for perception techniques applied to the automotive domain.

- Comprehensive Object Detector Study: Incorporating additional 3D object detectors from the state of the art aims to glean insights into the compatibility of different detectors that were originally designed for LiDAR data with RADAR data.
- Temporal Aggregation Investigation: The research aims to delve deeper into multi-frame aggregated networks specifically designed for temporal aggregation. The demonstrated benefits of scan accumulation in the proposed architecture prompt further exploration of its potential and optimization.
- Feature-Level Fusion Architectures: Novel approaches within the domain are contemplated, focusing on fusing information at the feature level within NNs. Attention mechanisms, such as cross-attention modules, are envisioned to facilitate the interaction between features from different data modalities.
- Optimization with Acceleration Frameworks: The proposal’s optimization journey involves leveraging DL acceleration frameworks and transitioning CPU processes to GPU implementations. This optimization is a necessary step toward integrating and testing algorithms within the real AD stack of the electric AV at the RobeSafe Research Group (University of Alcalá).

## Figures and Tables

**Table 1:** Summary of revisited state-of-the-art methods.

Method	Fusion Type	Description	Dataset	Year
Nobis et al. [9]	Data	Fusion in the image plane and extension in height of RADAR data.	nuScenes [6]	2019
CenterFusion [7]	Feature	Dual-branch backbone and feature fusion for detection.	nuScenes [6]	2021
RadSegNet [12]	Data	Fusion-then-detection in BEV. RADAR point cloud and image semantics projected.	Astyx [14]	2022

**Table 2:** Quantitative results of PointPillars-R with IoU thresholds (0.5, 0.25, 0.25) over the validation set of View of Delft dataset. Best results in **bold**.

	BEV			3D			AOS		
	**Easy**	**Med**	**Hard**	**Easy**	**Med**	**Hard**	**Easy**	**Med**	**Hard**
	**AP40 Car (IoU @ 0.5)**								
**1 frame**	29.63	**40.10**	**33.21**	24.20	30.42	**27.40**	20.14	**28.28**	**23.60**
**3 frames**	**32.78**	36.62	30.41	25.33	**30.52**	24.95	**21.52**	25.94	21.47
**5 frames**	31.69	32.31	26.44	25.85	28.77	21.13	21.06	21.02	17.07
	**AP40 Pedestrian (IoU @ 0.25)**								
**1 frame**	37.46	33.36	**30.61**	30.45	27.37	24.18	19.22	16.88	15.25
**3 frames**	**38.20**	**33.62**	29.85	**31.76**	**28.26**	24.88	**21.27**	**18.45**	**16.55**
**5 frames**	36.09	33.13	29.41	30.44	27.81	**24.97**	20.04	17.85	15.91
	**AP40 Cyclist (IoU @ 0.25)**								
**1 frame**	67.53	63.79	57.81	63.25	58.29	53.42	48.28	44.61	39.74
**3 frames**	71.53	66.19	59.94	66.88	61.50	55.40	51.71	46.71	42.26
**5 frames**	**76.41**	**70.12**	**63.60**	**73.15**	**66.97**	**60.31**	**58.22**	**53.51**	**47.56**

**Table 3:** Quantitative results of Painted PointPillars-R with IoU thresholds (0.5, 0.25, 0.25) over the validation set of View of Delft dataset. Best results in **bold**.

	BEV			3D			AOS		
	**Easy**	**Med**	**Hard**	**Easy**	**Med**	**Hard**	**Easy**	**Med**	**Hard**
	**AP40 Car (IoU @ 0.5)**								
**1 frame**	45.27	41.03	33.85	32.95	32.45	26.00	24.65	25.79	20.80
**3 frames**	51.41	39.59	32.87	43.05	33.56	**32.87**	33.55	26.23	**27.15**
**5 frames**	**62.59**	**51.95**	**43.16**	**47.21**	**39.64**	32.00	**33.83**	**30.20**	24.73
	**AP40 Pedestrian (IoU @ 0.25)**								
**1 frame**	39.62	34.90	31.29	32.37	28.64	25.31	20.38	18.35	16.17
**3 frames**	44.51	40.85	36.94	40.06	35.66	31.98	28.71	25.60	23.11
**5 frames**	**53.80**	**50.08**	**44.65**	**47.23**	**43.33**	**38.85**	**31.90**	**29.52**	**26.26**
	**AP40 Cyclist (IoU @ 0.25)**								
**1 frame**	75.76	71.01	64.25	68.27	62.28	55.76	46.75	41.94	36.95
**3 frames**	79.70	75.69	69.31	77.36	73.24	66.67	65.23	60.42	54.43
**5 frames**	**81.97**	**77.71**	**70.85**	**79.44**	**75.03**	**67.91**	**64.21**	**59.36**	**53.34**

**Table 4:** Inference time for YOLOv8-seg series.

Model	Preprocessing	Inference	Postprocessing	Total
**YOLOv8n-seg**	0.8 ms	1.7 ms	0.5 ms	3.0 ms
**YOLOv8s-seg**	0.7 ms	2.1 ms	0.5 ms	3.3 ms
**YOLOv8m-seg**	0.7 ms	3.9 ms	0.5 ms	5.1 ms
**YOLOv8l-seg**	0.7 ms	5.5 ms	0.5 ms	6.7 ms
**YOLOv8x-seg**	0.8 ms	6.8 ms	0.5 ms	8.1 ms

**Table 5:** Inference time for PointPillars-R and Painted PointPillars-R models.

Model	Dataset Version	Mean Inference Time	Std. Deviation Inference Time
**PointPillars-R**	1 frame	6.15 ms	0.27 ms
	3 frames	6.15 ms	0.25 ms
	5 frames	6.14 ms	0.28 ms
**Painted PointPillars-R**	1 frame	6.27 ms	0.29 ms
	3 frames	6.29 ms	0.30 ms
	5 frames	6.34 ms	0.31 ms

**Table 6:** Performance comparison between PointPillars and Painted PointPillars for RADAR (R) and LiDAR (L).

	AP40 Car	AP40 Ped.	AP40 Cyc.	mAP40@3D
**PointPillars-R (5f)**	28.77	27.81	66.97	41.18
**Painted PointPillars-R (5f)**	39.64	43.33	75.03	52.67
**PointPillars-L**	70.80	56.79	80.59	69.40
**Painted PointPillars-L**	75.07	62.04	82.32	73.14

**Table 7:** Ablation study for the pipeline of Painted PointPillars-R, reflecting the contribution of every element of the system individually.

Baseline	+ Painting	+ Cluster Refinement	+ 3 Frames	+ 5 Frames	mAP
√					38.69
√	√				40.86
√	√	√			41.12
√	√	√	√		47.48
√	√	√		√	52.67

## Data Availability

In this manuscript, View of Delft dataset has been used. It can be accessed via request to their original authors. The procedure and elegibility to use data can be found at their official website (https://tudelft-iv.github.io/view-of-delft-dataset/, accessed on 27 December 2023).

## Abbreviations

The following abbreviations are used in this manuscript:

ACC	Adaptive Cruise Control
AD	Autonomous Driving
ADAS	Advanced Driver Assistance Systems
AEB	Automatic Emergency Braking
AOS	Average Orientation Similarity
AV	Autonomous Vehicle
BEV	Bird’s Eye View
DBSCAN	Density-Based Spatial Clustering for Applications with Noise
IoU	Intersection over Union
LDW	Lane Departure Warning
LiDAR	Lidar Detection and Ranging
mAP	Mean Average Precision
NMS	Non-Maximum Suppression
NN	Neural Network
RADAR	Radio Detection and Ranging
VOD	View of Delft dataset
VRU	Vulnerable Road User
